# *Mycobacterium bovis* Bacille Calmette-Guérin as a Vaccine Vector for Global Infectious Disease Control

**DOI:** 10.1155/2011/574591

**Published:** 2011-05-24

**Authors:** Kazuhiro Matsuo, Yasuhiro Yasutomi

**Affiliations:** ^1^R & D Department, Japan BCG Laboratory, 3-1-5 Matsuyama, Kiyose, Tokyo 204-0022, Japan; ^2^Tsukuba Primate Research Center, National Institute of Biomedical Innovation, 1-1 Hachimandai, Tsukuba, Ibaraki 305-0843, Japan; ^3^Department of Immunoregulation, Mie University Graduate School of Medicine, Tsu City, Mie Prefecture 514-8507, Japan

## Abstract

*Mycobacterium bovis* bacille Calmette-Guérin (BCG) is the only available vaccine for tuberculosis (TB). Although this vaccine is effective in controlling infantile TB, BCG-induced protective effects against pulmonary diseases in adults have not been clearly demonstrated. Recombinant BCG (rBCG) technology has been extensively applied to obtain more potent immunogenicity of this vaccine, and several candidate TB vaccines have currently reached human clinical trials. On the other hand, recent progress in the improvement of the BCG vector, such as the codon optimization strategy and combination with viral vector boost, allows us to utilize this bacterium in HIV vaccine development. In this paper, we review recent progress in rBCG-based vaccine studies that may have implications in the development of novel vaccines for controlling global infectious diseases in the near future.

## 1. Introduction

*Mycobacterium bovis *bacille Calmette-Guérin (BCG) is the only licensed vaccine that has substantially helped controlling tuberculosis (TB) for more than 80 years. This vaccine affords ~80% protection against TB meningitis and miliary TB in infants and young children [1]. However, the BCG-induced protective effects against pulmonary diseases over all ages are variable; the escalation of the worldwide TB epidemic is evidence that the vaccine does not work well to prevent pulmonary TB [2]. Recently, studies on the advanced molecular biology and genomics of mycobacteria have revealed that the BCG genome has various mutations and deletions compared with the original virulent strain of *Mycobacterium tuberculosis* and *M. bovis* [3]. Interestingly, there are substantial differences in the genomic DNA even among BCG substrains [4, 5] that can cause biological differences in the population of BCG vaccines.

Since a host-vector system in mycobacteria was developed in 1987 [6], recombinant BCG (rBCG) technology has been extensively applied in the development of vaccines against a variety of infectious diseases, including bacterial, viral, and parasitic infections in addition to TB [7, 8]. BCG is attractive as a vaccine vector because of its extensive safety record in humans, heat stability, low production cost, induction of long-lasting type 1 helper T cell (Th1) immunity, CD8^+^ T-cell triggering, adjuvant activity, usability in newborns and its mucosal immune induction by oral administration. Taking the current situation of serious epidemics of emerging and reemerging diseases mainly in developing African and Asian countries into account, a new global vaccine should be affordable in such areas. Therefore, the low price and heat stability of BCG-based vaccines would be desirable. In this paper, we review various efforts to develop novel BCG vector-based vaccines mainly for controlling TB and HIV/AIDS.

## 2. Immunological Properties of BCG Vector

**Figure 1:**
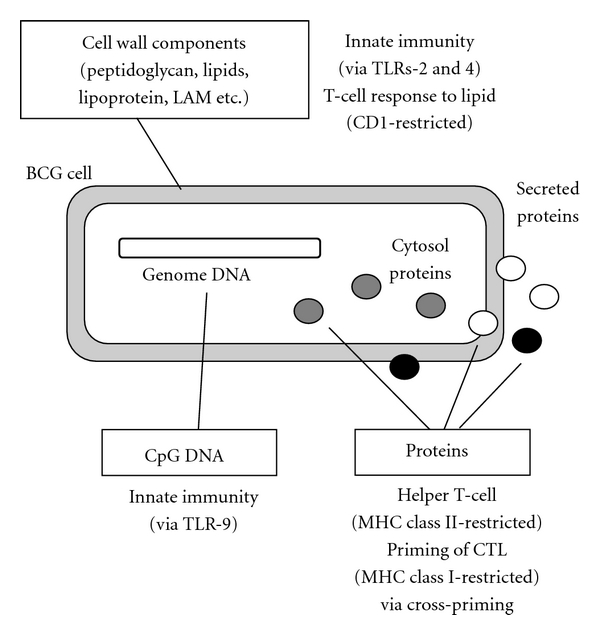
Outline of immune responses by BCG. Both innate immunity via TLRs and antigen-specific immunity via MHC- or CD1-restricted antigen presentation to T cells are induced by various BCG cell components.

The immune responses induced by BCG are outlined in Figure 1. The most characteristic response to BCG is the induction of innate (nonspecific) immunity by cell wall components through toll-like receptors (TLRs) 2 and 4 on dendritic cells and macrophages [9]. After phagocytosis, BCG is degraded by lysosomal enzymes, and the processed antigen can be presented to the host immune system via various pathways. DNA fragments containing the CpG motif may activate innate immunity via the TLR9 route [10]. Lipids such as mycolic acid presented by CD1 stimulate CD1-restricted CD8^+^ T cells [11]. Protein antigens, such as antigen 85 complex produced by BCG, induce Th1 response through presentation by major histocompatibility complex (MHC) class II. This pathway is the major route of BCG-induced responses and is indispensable for protective immunity against *M. tuberculosis* infection via protective cytokine interferon (IFN)-*γ* production. On the other hand, the processing and presentation of protein antigens via the MHC class I pathway are also elicited in the BCG-infected antigen presenting cell (APC). As reported by Goonetilleke et al. [12], immunizing BCG-sensitized animals with recombinant vaccinia virus MVA expressing antigen 85A greatly enhances the MHC class I-restricted CTL response against antigen 85A, indicating that BCG priming could be a novel type of prime-boost vaccine. This immunological feature of BCG vector allows its application in vaccines against chronic viral infectious diseases such as HIV/AIDS. In addition, the strong Th1 induction by BCG would be favorable to aid the maturation and maintenance of CTL [13]. Thus, the BCG vector is expected to induce effective cell-mediated immunity against a targeted antigen.

## 3. TB Vaccine

### 3.1. Background of the Global TB Epidemic

TB kills 1.7 million people worldwide each year; someone dies from TB every 19 seconds [14]. Although the TB treatment protocol was established a long time ago, the recent increase of multidrug-resistant *M. tuberculosis* infection has generated a serious situation. New vaccines are urgently needed to eliminate TB as a public health threat and should be a major global public health priority. TB is a disease that is spread from person to person through the air. Furthermore, the terrible synergy between TB and HIV makes this disease even more dangerous, especially in sub-Saharan African countries. For instance, according to the World Health Organization's (WHO) Global TB report 2010 [14], South Africa had nearly 400,000 new TB cases in 2009 with an incidence rate of an estimated 806 cases per 100,000; TB is one of the leading causes of death in both adults and children of this country. The case fatality rate has increased from 3% in 1993 to 24.3% in 2007. A major reason for the increased fatality rate is South Africa's concurrent HIV epidemic. The prevalence of HIV infection in South Africa in 2009 was approximately 7%, which has been decreasing as a result of various efforts toward prevention. TB is a common opportunistic infection among people living with HIV, and 60% of new TB cases occurred in persons who were also infected with HIV in 2009 [14]. We can observe similar critical situations in the countries surrounding South Africa. Regarding the vaccination, such situation has raised concerns about the safety of using BCG vaccine in HIV-infected infants because between 10 and 30% of pregnant women are HIV infected in many sub-Saharan African countries.

### 3.2. Current Efforts toward New TB Vaccine Development

The global plan to stop TB 2011–2015 report [15] offers 7 objectives as follows: (i) to maintain a robust TB vaccine pipeline by supporting research and discovery, (ii) to conduct research to identify correlates of protection and preclinical studies to assess new TB vaccine candidates, (iii) to ensure the availability of vaccine production capacity by expanding manufacturing facilities for TB vaccines, (iv) to build capacity for large-scale clinical trials (phases II and III) of TB vaccine candidates at field sites in TB-endemic countries, (v) to conduct phase I, II, and III clinical trials of TB vaccine candidates, (vi) to develop delivery, regulatory, and access strategies for new TB vaccines, (vii) to build support for TB vaccine development and uptake through advocacy, communications, and resource mobilization. All these objectives are important to realize new TB vaccine development.

The main goal of vaccine development in the Global Plan to Stop TB 2006–2015 is for 2 vaccines to be in proof-of-concept trials by 2010 and that 1 new and safe vaccine is available by 2015. As of 2009, 12 TB vaccine candidates had entered clinical trials. Of these, 9 are still being tested (Table 1): 5 are in phase I clinical trials, 2 are in phase II trials, and 2 are in phase IIb proof-of-concept trials [15]. One vaccine has produced estimates of safety and effectiveness in a targeted HIV-infected population. At least 6 TB vaccine candidates are in preclinical development, and at least 21 additional next-generation candidates are in the vaccine discovery phase [15]. As mentioned earlier, the current BCG vaccine has limited and variable effectiveness against TB. Therefore, the first choice of strategy may be improving BCG by using recombinant DNA technology even though it may imply safety issue of vaccination in HIV-infected individuals. Overproduction against a protective antigen of TB in BCG (rBCG30) exhibited enhanced immunogenicity in humans [16]. Moreover, the expression of the listeriolysin gene in BCG (rBCG/*hly^+^*::Δ*ureC*) is proven to be more potent in the induction of TB-specific cellular immune responses [17]. Another strategy for improving BCG vaccines is boosting BCG immunity with protein [18, 19] or viral vector vaccine such as modified vaccinia virus Ankara (MVA) strain [20] and adenovirus type 35 [21]. BCG-prime and recombinant MVA-antigen 85A boost regimen [22] exhibited efficient immune responses in humans and have entered the first phase IIb trial in newborns. Furthermore, a combination of such strategies in which 3 major antigens are overproduced and the perfringolysin gene is incorporated into BCG and boosted with a recombinant adenovirus vaccine has been developed [23]. However, it is unknown whether such strategies are relevant for developing vaccines that are effective against adult pulmonary TB. It is necessary to test whether these candidate vaccines effectively induce mucosal immunity and protect against lung disease.

## 4. HIV/AIDS Vaccine

### 4.1. Background of the Global HIV Epidemic

In 2009, there were an estimated 2.6 million people who became newly infected with HIV. This is more than 21% less than the estimated 3.2 million who became infected in 1997, the year in which annual new infections peaked. In 33 countries, the incidence of HIV has decreased by more than 25% between 2001 and 2009; 22 of these countries are in sub-Saharan Africa. This trend reflects a combination of factors including the impact of HIV prevention efforts and the natural course of HIV epidemics [24]. 

Although highly activated antiretroviral therapy apparently contributes to control HIV replication in infected individuals [25], several problems remain to be resolved. These problems include: (i) the following viral load recovers soon after the interruption of treatment; (ii) chronic toxicities cause abnormalities in lipid metabolism and mitochondria; (iii) drug-resistant viruses increase during long period of treatment; (iv) long-term treatment carries a risk of carcinogenesis [26]; (v) expensive drugs are still difficult to access in developing countries. Even in developed countries, the high cost of antiretroviral drugs produces a sense of impending crisis in public health policy [27]. In such circumstances, although the rate of new infections with HIV-1 is gradually decreasing, an effective preventive vaccine is still urgently needed to stem further spread of the virus [28]. Even though considerable recent progress has been made in the development of an HIV vaccine [29, 30], the immune correlate of viral protection is not fully elucidated due to the complicated interaction of viral, immunological, and genetic factors [31, 32]. Since it is known that some populations of HIV-1-infected people do not present disease progression when HIV-1 replication is regulated by host immunity [33, 34], targeted vaccine immunogens are designed to closely mimic the long-lasting protective immunity induced in the long-term human survivors of natural infection [35, 36]. Due to safety issues, a live-attenuated HIV vaccine is not practical. This inevitably led the trend of HIV vaccine development to component- and vector-based vaccines.

### 4.2. Current Trends in HIV/AIDS Vaccine Research

The first large-scale efficacy trial of an HIV/AIDS vaccine was conducted by a US company, Vaxgen Co., in which a genetically engineered surface envelope (Env) glycoprotein, gp120, vaccine was tested in humans. Although the vaccine was targeted toward inducing effective virus-neutralizing antibodies, the phase III efficacy trial revealed its ineffectiveness [37, 38]. The failure of the gp120 vaccine changed the trend of HIV/AIDS vaccine research from an antibody-targeted strategy to a cell-mediated immunity-targeted strategy. Because HIV-1 causes chronic infection due to its cell-associated features, cellular immunity especially virus-specific cytotoxic T lymphocyte (CTL) should be a more important arm of the host immune system. Indeed, immune deficiency virus-specific cell-mediated immunity has been suggested to effectively control viral replication during the natural course of viral infections [39–41]. Based on these findings, various vaccine modalities, including live viral vectors and DNA vaccines, have been used to elicit strong CTL and Th1 type responses in nonhuman primate models. Although single-vaccine delivery systems sometimes exhibit insufficient immune responses, boosting with viral vector vaccines such as vaccinia virus [40, 41], adenovirus [42, 43], and Sendai virus [44] in DNA-primed individuals strongly amplified CTL responses and resulted in the effective control of simian immunodeficiency virus (SIV) replication. Among such viral vectors, adenovirus type 5 (Ad5) had the strongest CTL enhancement effect, and the DNA-prime and recombinant Ad5 boost vaccine strategy is recognized as the most promising. However, in 2007, Merck Co. reported that a recombinant Ad5 vaccine expressing HIV-1 Gag, Pol, and Nef antigens did not demonstrate any protective efficacy in a phase IIB clinical trial [45]. Surprisingly, the vaccinated group exhibited a significantly higher HIV-1 infection rate than the placebo group [45], suggesting that the recombinant Ad5 immunization may have some unknown effect in enhancing HIV-1 infection. Thus, we were aware that T-cell vaccine approaches may involve certain risks and limitations; this paradigm appears to have reached an impasse.

In September 2009, there was ground-breaking news that the RV144 large-scale efficacy trial in Thailand demonstrated a partial effect of reducing HIV-1 infection rate in the recipients of ALVAC (canarypox)/gp120 prime-boost vaccine [46]. Although the results demonstrated limited effects, they demonstrated the possibility of preventing HIV infection with the active immunization for the first time. Furthermore, although there was no apparent correlation between protection and virus-specific cellular immune response or neutralizing antibody levels in the vaccinees, more detailed analyses of the total host responses are expected in the future. Taking the vaccine formulation with the gp120 protein boost into account, some antibody-mediated reactions may be involved in this partial protection. On the other hand, a new T-cell-targeted vaccine also demonstrated protective efficacy in a macaque study in the same year. A rhesus cytomegalovirus-vectored vaccine expressing SIV Gag, Rev-Tat-Nef, and Env persistently infected rhesus macaques, primed, and maintained robust SIV-specific CD4^+^ and CD8^+^ effector memory T-cell responses in the absence of neutralizing antibodies [47]. The report suggests that T cell vaccines may have greater potential than previously estimated. Although the importance of broadly neutralizing antibody production would not change despite tremendous difficulties, cellular immunity-targeted candidate vaccines should be also clinically tested for proofs of concept.

### 4.3. BCG-Vectored HIV Vaccine

The most practical advantage of the BCG vector is its high safety. In addition to being effective at inducing protective immunity, an HIV-1 vaccine regimen must be shown to be safe, affordable, and compatible with other vaccines before it can be considered promising [39]. In this respect, vectors that have already been used in humans without serious complications and with low cost should be utilized for HIV vaccines. BCG is a unique live vaccine vector because of its easy antigen delivery to the professional APC to be presented to T cells. Therefore, this bacterium is expected to be an important vector for HIV vaccine development.

At the early stage of rBCG research in the 1990s, Aldovini and Young [48] demonstrated immunogenicity of rBCG against genetically engineered HIV-1 antigens in mice. We independently worked on an rBCG-vectored anti-HIV vaccine simultaneously. First, we demonstrated effective cellular immune induction against SIV Gag antigen by the rBCG vector in rhesus macaques [49, 50]. Furthermore, we cloned an extracellular *α* antigen (antigen 85B) gene from both BCG [51] and *Mycobacterium kansasii *[52], and established a foreign antigen secretion system in mycobacteria [53]. Based on this system, we extensively evaluated several rBCG constructs for candidate HIV vaccines and reported that an rBCG-HIV vaccine could induce protective humoral immune responses in guinea pigs [54]. These studies suggest that rBCG-based vaccines are feasible as AIDS vaccines. However, the CTL activity did not reach protective levels with a single injection of rBCG-HIV vaccine in the macaque model. To overcome the low immunogenicity of the rBCG vaccine in CTL induction, we utilized various strategies for enhancing the immune potential of the BCG vector.

### 4.4. Prime-Boost Regimen for Enhancing Immune Responses

The first strategy by which we tried to improve the potential of the rBCG-HIV vaccine was the use of a safe recombinant viral vector for a booster vaccine. With respect to safety, traditional live vaccines, which have been administered safely to both the healthy and the HIV-infected individuals, may be the vectors of choice for HIV-1 vaccines. To fully take advantage of the benefits of such traditional vaccines in the development of anti-HIV vaccines, we studied BCG Tokyo 172 strain and the replication-deficient vaccinia vaccine strain DIs [55, 56] both of which have been shown to be nonpathogenic when inoculated into immune-deficient animals as live recombinant vaccine vehicles [57]. The vaccinia virus DIs have been tested clinically as a smallpox vaccine in Japanese infants and proved to be quite safe. We chose this highly attenuated virus as a booster vaccine vector and constructed recombinant DIs (rDIs) expressing the HIV *gag* [58] or SIV *gag-pol* gene [59]. Both rDIs constructs were found to be effective in eliciting HIV- or SIV-Gag-specific immunity in mice. When they were administered as a booster antigen after priming with an SIV-DNA vaccine, the cellular immunity to SIV Gag was greatly enhanced [59]. In brief, we tested a new combination regimen: priming with rBCG-SIV Gag followed by boosting with rDIs-SIV Gag.

**Figure 2:**
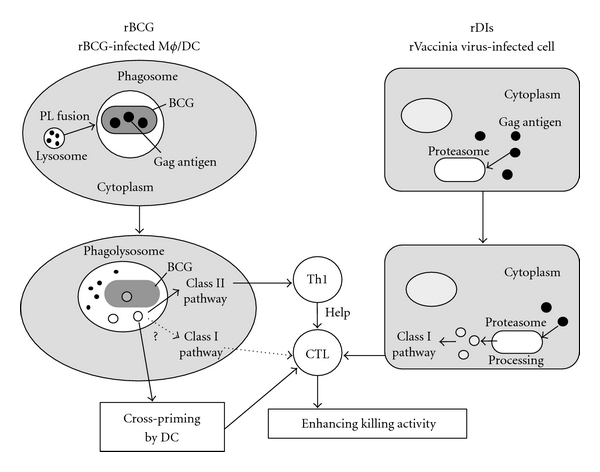
A possible mechanism of effective Gag-specific cell-mediated immunity induction with the rBCG/rDIs prime-boost vaccine. Abbreviations: DC, dendritic cell; M*ϕ*, macrophage; PL, phagosome-lysozome; Th1, type 1 helper T cell; CTL, cytotoxic T lymphocyte.

In the macaque study, we found that BCG/DIs vaccination induced a long-lasting and effective cellular immunity that was able to control a highly pathogenic virus SHIV C2/1 [60], after mucosal challenge [61]. A possible mechanism of effective Gag-specific cell-mediated immunity is shown in Figure 2. The strong Th1 response induced by the BCG vector may contribute to eliciting the Gag-specific CTL response. How these immune inductions are correlated with protective efficacy requires further investigation. In this study, the BCG/DIs vaccination developed high levels of cellular immunity in the macaques that were protected against the loss of CD4^+^ T lymphocytes with reduced viral RNA levels after virus challenge. Furthermore, the BCG/DIs group showed no evidence of clinical diseases or mortality after viral challenge during the 1-year observation period [61]. These results suggest that the BCG/DIs prime-boost regimen might be a potential candidate for an effective and safe anti-HIV vaccine. Recent studies in macaques subjected to BCG/Ad5 [62] and BCG/MVA [63] regimens strongly support the effectiveness of the BCG vector. In the latter study, a hemolysin-expressing BCG strain, which was devised for more efficient antigen presentation to the CTL precursor, elicited a robust and broad range of HIV-1 specific T-cell responses along with recruitment of multiple T-cell clonotypes into the memory pool.

### 4.5. Codon Optimization Strategy

The major issue with BCG vehicle vaccines is the low expression level of the foreign antigen gene in BCG cells. In general, sufficient levels of foreign antigen-specific immune responses are obtained with high doses of rBCG between 10- and 100-fold greater than that needed for a practical dose against TB in humans [54]. This is considered the main limitation for the clinical use of rBCG-based vaccines. To address this substantial issue, we applied a codon optimization strategy for foreign genes in the rBCG system to increase its expression level. The aims of the study were to increase the immunogenicity of the foreign antigen, decrease inoculation dosages as small as the conventional BCG vaccine against TB, avoid adverse reactions, prevent possible association with Th2-type immune responses, and ward off the exacerbation of retroviral infections.

First, we determined the in vitro effects of codon optimization of the HIV gene in rBCG. Although the effect of codon optimization in mammalian cells is well documented [64–66], its effect in rBCG vehicle had never been fully elucidated. We targeted the HIV-1 *gag p24* gene as a model antigen to clarify the effect of codon optimization in the rBCG system. A specially designed synthetic p24 gene consisting of mycobacterial-preferred codons resulted in an increase in their GC content from 43.4% to 67.4%. Furthermore, codon-optimized rBCG was generated without any detectable changes in its characters including the growth rate. This rBCG exhibited a dramatic increase in Gag p24 antigen production approximately 40-fold greater than the nonoptimized rBCG. Moreover, we successfully obtained data regarding the enhancement of immune responses in codon-optimized rBCG-immunized mice [67]. Inoculation of mice with a single low dose of the codon-optimized bacteria elicited effective cellular immunity. In the ELISPOT assay, the number of Gag-specific IFN-*γ* spot-forming cells elicited by codon-optimized rBCG was significantly greater than that elicited by non-optimized recombinants [67]. These cellular immune responses would decrease if the CD8^+^ T cells were depleted. The results also suggest that effective MHC-class I-restricted CTL responses are inducible by vaccination with codon-optimized rBCG. Furthermore, Gag-specific lymphocyte proliferative responses were also detected in the codon-optimized rBCG-immunized mice [67]. 

We also applied this strategy to an SIV Gag construct and successfully generated an rBCG harboring the codon-optimized SIV *gag* gene with an expression 10-fold greater than that of the native *gag* gene. In the macaque study, compared with a native *gag* gene construct, a low-dose (10^6^ bacilli) injection of this construct induced optimal priming of Gag-specific CD4^+^ and CD8^+^ T cells and prolonged the maintenance of memory T-cell response after vaccinia DIs boost [68]. These results imply that the quality of the priming vaccine is a critical factor for inducing a desirable immune response against immunodeficiency viruses. Thus, the codon optimization strategy should generally be applied to other foreign genes in rBCG-based vaccine development.

## 5. Vaccine for Other Infectious Diseases

There were various candidate rBCG vaccines targeting infectious diseases other than TB or HIV. Stover et al. [69] reported that the rBCG system would be useful in Lyme disease vaccine development; the vaccine incorporated with the surface protein of *Borrelia burgdorferi* first reached clinical phase I trials. However, the vaccine was rejected due to its low antibody production response [70]. Two groups [71, 72] applied rBCG in malaria vaccine development and demonstrated efficacy in a mouse model. Malaria is recognized as one of the three major infectious diseases as well as TB and AIDS. Although there is a long history of malaria vaccine development, we have not seen any licensed vaccine. The strategy to induce cellular immunity against conserved antigens using BCG vector could be effective to overcome substantial difficulties in producing vaccine due to antigenic diversity and unique life cycle of this parasite. In addition, BCG vector was tested for vaccine discovery against some viral diseases. A rBCG expressing the measles virus nucleoprotein demonstrated protection against measles virus pneumonia in macaques [73]. Furthermore, we demonstrated that a rBCG with a single hepatitis C virus (HCV) NS5 CTL epitope into antigen 85B induced HCV-specific CTL response in mice [74]. HCV is recognized as one of the major infectious pathogens of which the global infection rate is ~3%. Although the priority for preventive HCV vaccine development has become lower because of the remarkable progress in the treatment, BCG vector of targeting CTL induction may have implication for therapeutic vaccine against this disease. All these candidates at the early stage of rBCG study could not proceed to further development stages at those times. The rBCG-based vaccine development for these diseases should be reconsidered because the advanced technology that enhances the potential of BCG vectors has become currently available.

## 6. Conclusion and Future Perspective

As described in Section 3, several rBCG-based candidate vaccines are currently being evaluated for the development of TB vaccines. Such human trials would provide a greater insight into the paradigm of immune correlation in *M. tuberculosis* infection. In addition, the application of the codon optimization strategy enables us to utilize this bacterial vector as a primer of a heterologous prime-boost regimen for a preventive HIV vaccine. These results could suggest that the BCG vector is possible divalent vaccine controlling both TB and HIV/AIDS with a single construct; such study may help resolve the serious public health problem in the sub-Saharan African countries in which both diseases are highly prevalent [14].

Another potential outcome is the utility of the BCG vector for infant vaccines. One of the largest advantages of rBCG vaccines is their applicability to newborns. Because BCG as a TB vaccine is integrated into the expanded program on immunization in many countries, we have the earliest chance to immunize newborns with BCG within 3 months of birth before they are exposed to a variety of infectious pathogens. Substituting the current BCG with a novel rBCG vaccine possessing protective antigens against pathogens that cause serious diseases in infants, such as severe diarrhea and respiratory diseases, could be effective in developing countries. Such vaccine concepts should be also tested in appropriate animal models before they are tested in humans. Thus, after much trial and error in the last 2 decades, rBCG-based vaccines may contribute to the control of global infectious diseases in the near future.

## Figures and Tables

**Table 1:** Summary of candidate TB vaccines in clinical trials 2009. Nine candidate preventive TB vaccines are currently in clinical phases.

Status	Products	Product description	Sponsor
Phase IIb	MVA85A/AERAS-485	Vaccinia virus MVA	OETC/AERAS
Phase IIb	AERAS-402/Crucell Ad35	rBCG/adenovirus 35	Crucell/AERAS

Phase II	Hybrid-I + IC31	Ag85B/ESAT6 + adjuvant	SSI/TBVI
Phase II	M72	Fusion protein + adjuvant	GSK/AERAS

Phase I	AdAg85A	adenovirus 5/Ag85A	McMaster Univ.
Phase I	VPM 1002	rBCG/listeriolysin::ΔureC	Max Planck/TBVI
Phase I	Hyvac 4/AERAS-404	Fusion protein + adjuvant	SSI/Sanofi/AERAS
Phase I	RUTI	Fragmented Mtb cell	Archivel Farma
Phase I	Hybrid-I + CAF01	Ag85B/ESAT6 + adjuvant	SSI

Abbreviations in the sponsors: AERAS, AERAS Global TB Vaccine Foundation; GSK, GlaxoSmithKline; OETC, The Oxford-Emergent Tuberculosis Consortium Ltd.; SSI, Staten Serum Institute; TBVI, Tuberculosis Vaccine Initiative.
